# Optimization-Based Approaches for Minimizing Deployment Costs for Wireless Sensor Networks with Bounded Estimation Errors

**DOI:** 10.3390/s21217121

**Published:** 2021-10-27

**Authors:** Chiu-Han Hsiao, Frank Yeong-Sung Lin, Hao-Jyun Yang, Yennun Huang, Yu-Fang Chen, Ching-Wen Tu, Si-Yao Zhang

**Affiliations:** 1 Research Center for Information Technology Innovation, Academia Sinica, Taipei 115, Taiwan; garry0325@citi.sinica.edu.tw (H.-J.Y.); yennunhuang@citi.sinica.edu.tw (Y.H.); chingwe1@asu.edu (C.-W.T.); 2 Department of Information Management, National Taiwan University, Taipei 10617, Taiwan; flin@ntu.edu.tw (F.Y.-S.L.); D09725003@ntu.edu.tw (Y.-F.C.); R07725051@ntu.edu.tw (S.-Y.Z.)

**Keywords:** Lagrangian Relaxation, network deployment, pearson correlation, wireless sensor networks (WSNs), XGBoost

## Abstract

As wireless sensor networks have become more prevalent, data from sensors in daily life are constantly being recorded. Due to cost or energy consumption considerations, optimization-based approaches are proposed to reduce deployed sensors and yield results within the error tolerance. The correlation-aware method is also designed in a mathematical model that combines theoretical and practical perspectives. The sensor deployment strategies, including XGBoost, Pearson correlation, and Lagrangian Relaxation (LR), are determined to minimize deployment costs while maintaining estimation errors below a given threshold. Moreover, the results significantly ensure the accuracy of the gathered information while minimizing the cost of deployment and maximizing the lifetime of the WSN. Furthermore, the proposed solution can be readily applied to sensor distribution problems in various fields.

## 1. Introduction

Wireless sensor networks (WSNs) are used worldwide; approximately 500 billion devices in various industries are connected to the Internet. Applications include manufacturing, e-commerce, energy, surveillance, and environmental detection [1,2]. Sensors access information through the network to provide numerous innovative services. The diverse requirements of services are satisfied for networking between people, between people and machines, or even between machines for purposes ranging from residential to social communication [3]. Thus, humans and things are beginning to use the Internet to monitor air quality, temperature, landslides, and other data [4]. Placing a sensor at each point requiring data collection may cause sensor redundancy, resulting in a substantial wastage of resources [5,6]. However, reducing the number of deployed sensors may lead to acquisition of insufficient or incorrect information [7]. It is a trade-off for network planning and operation. For large-scale WSNs, system deployment designers must analyze relevant trade-offs to develop a protocol extending the lifetime of WSNs by improving energy efficiency and collecting data accurately. In this paper, the proposed sensor deployment strategies ensure the accuracy of the gathered information while minimizing the cost of deployment and maximizing the lifetime of the WSN. Based on previous studies as mentioned in [8], a preliminary analysis was performed for several methods of balancing WSN energy consumption. The variable-range transmission power control method optimizes traffic distribution by deploying sensors which are inexpensive compared to transmission costs. The mobile-data-sink deployment and multiple-data-sink deployment methods both adjust the position of the data sink in the network. Nonuniform initial energy assignment and intelligent sensor or relay deployment are also methods for reducing power consumption in WSNs [3].

**Figure 1:**
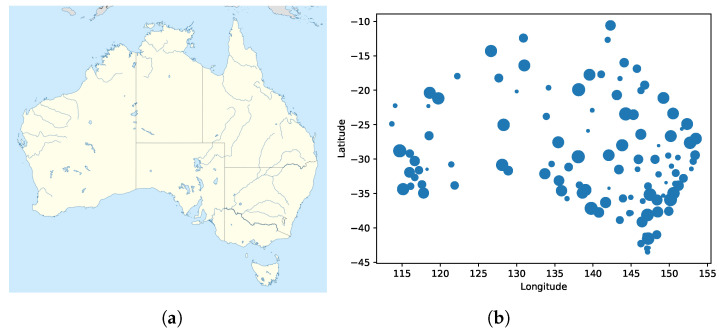
Australia map and the locations of the observation sites. (**a**) Map of Australia. (**b**) Locations of the observation sites.

This paper proposed deploying WSNs to provide services with sufficient lifetime and reduced cost and overall energy consumption. In particular, large-scale applications were investigated. By analyzing thermal sensor data from related experiments, we found that the temperature measurements of many sensors are correlated. The Australian Climate Observation Reference Network-Surface Air Temperature (ACORN-SAT) dataset includes consistent and uniform daily temperature records from 112 observation sites beginning in 1910. Figure 1a illustrates the Australia contour and Figure 1b presents all the sites; larger circles indicate a site with higher costs. The use of additional sensors increases the total amount of data. It increases the likelihood that similar data are collected between sensors. Excessive data are redundant and reduce processing efficiency. Therefore, a mathematical model was formulated to estimate the temperature values of locations without installed sensor nodes. In this paper, thermal sensors (for temperature data) were selected because they are representative of many other applications, such as in heating products, pipe temperature measurements for flowing liquids (e.g., oil or chemical products), smart homes, refrigerator temperatures, and large-scale environmental monitoring. The proposed method compared with previous works is called error-bound satisfaction and ensures the quality of the results. Performance metrics are used to evaluate results and ensure that estimation error values are within the system’s tolerance of the average estimation error. By guaranteeing the accuracy of the estimation systematically, the lifetime of the deployed WSN is sufficient to meet the requirements for controlling the sensors during operations. Sensing and installation costs are fixed during sensor installation. The primary contribution of this paper is to minimize deployment costs within given error bounds. Then, the cost minimization methods for WSNs meet numerous metrics. The proposed WSN deployment strategies can minimize cost and maintain accuracy within the system’s average estimation error threshold. This work combines both theoretical and practical considerations to minimize the deployment cost of temperature sensors. The proposed strategies are readily applied to sensor distribution problems in various fields.

Research Scope:

- Minimizing the deployment cost of temperature sensors.
- Data used in this research is from the Australian Climate Observation Reference Network-Surface Air Temperature.
- Data ranges from 2008–2019.
- Expected to significantly reduce the deployment cost while maintaining the error under threshold.

## 2. Related Work

WSN quality of service is measured in the literature by considering coverage, connectivity, network lifetime, and network deployment costs [9]. Several mathematical models, algorithms, and heuristics also have been proposed to solve other problems such as region of interest monitoring, intruder detection, and energy efficiency management in various applications. This section briefly identifies the shortcomings of these methods. It derives related sensor node deployment strategies depending on numerous factors for the system design in network planning and operations. Some strategies include placing sensors to minimize the number of sensors, reducing cost, and ensuring the accuracy of the data collected results.

### 2.1. Correlation-Aware Deployment Methods

The sensitivity of the estimation error depends on either the static sensor deployment or dynamic adjustment strategies. Methods of identifying the most informative sensor were proposed in [10]. Roy et al. assumed that a set of data snapshots could characterize the monitored phenomenon. To reconstruct the data with the required accuracy, a method for identifying optimal sensor locations was formulated. However, to handle both stationary and nonstationary fields, two optimization models were proposed. For both deployment problems, an iterative solution algorithm was proposed to obtain a sensor deployment strategy. Although the input was assumed to be perfect, errors may exist in the simulated data based on this assumption [11].

Additionally, for monitoring spatial phenomena such as temperatures in indoor or preconfigured environments, we can assume that the collection of data is possible in the predeployment phase. Krause et al. defined the quality of a given topology using the concept of mutual information to choose the best location for sensors using a Gaussian is the variation of this method [12]. The solution was a polynomial algorithm defined using the submodules of mutual information after formalizing the problem. The paper was extended by [13], considering not only the coverage area but also the connection cost; the qualities of the links were assumed to be Gaussian. Based on the results of the temperature measurements, the Gaussian approach was unsuitable [13]. A new temperature measurement and prediction method was designed using mathematical programming techniques.

A classic problem of measurement is the estimation of the data collected by a small set of deployed sensors to reduce costs. In [14], Ranieri et al. proposed a greedy heuristic algorithm to solve the related problem of perception deployment by considering a general form and studying its mathematical characteristics. Simulations were conducted to prove that the algorithm can solve the problem in a short time and can provide an approximate optimal solution. In [15], a perception topology was defined to select active sensors and inactivate other sensors. Liaskovitis et al. considered an already-deployed sensor network and proposed an algorithm to define the network. To determine whether a sensor remains active, they estimated changes in the sensed phenomenon online. By contrast, in this paper, we propose that offline selection of the sensing points is made during the network planning stage.

Furthermore, analysis of sensing data is essential. Machine learning and multiple linear regression models were compared for remote sensing data [16]. Forkuor et al. proposed methods including multiple linear regression, random forest regression, support vector machine, and stochastic gradient boosting and compared the performance metrics. The results revealed that multiple linear regression had the predicting ability. However, this method is limited by the relationship between dependent and independent data variables. The use of artificial intelligence and machine learning has become more prevalent, leading to an increase in input sensor data. However, some input data may not be useful for the model. In [17], Yan et al. used multiple linear regression to predict results with a small number of useful variables [17]. The correlation analysis can identify the most correlated data to predict missing sensor data with low error [18].

In [19], Ma et al. derived a sensor deployment scheme that eliminates vacancy points that cannot be estimated, achieving low sensor density and guaranteeing bounded estimation error. Ma et al. compared various spatial patterns, including the equilateral triangle, square, and regular hexagon. They concluded that an equilateral triangle pattern was the best deployment strategy. In [20], Kim et al. proposed an efficient deployment scheme for a surveillance sensor network incorporating the event occurrence rate. The scheme aimed to minimize the number of sensors deployed in a large-scale WSN and satisfy the target probability of detection. Their proposed scheme reduced the total number of sensors by 10% to 40%. In [21], Han et al. established a deployment strategy for underwater acoustic sensors. Their strategy considered network distribution in a three-dimensional environment. They simulated numerous deployment schemes and demonstrated that a tetrahedral scheme had better overall performance in reducing error.

### 2.2. Sensor Deployment Applications

The applications of WSNs are versatile. Ramesh et al. proposed a system of pore pressure transducers, dielectric moisture sensors, and movement sensors to detect landslides in real time [22,23]. The system had numerous sensor columns distributed over an area of interest. Moreover, their work included a complete architecture of the physical sensor columns and the backend software service. Huang et al. proposed a fiber-optic sensing system capable of monitoring debris flows [24]. The system included a light source, a data logger, a four-port coupler, and four fiber Bragg grating accelerometers. The results revealed that the proposed fiber-optic system outperformed conventional sensing systems and had high reliability; the system’s performance was promising for monitoring natural disasters. Marin-Perez et al. proposed a building automation system using the PLUG-N-HARVEST architecture that uses Internet of things (IoT) to achieve reliable security and intelligent management [25]. The automated building had low energy consumption. Moreover, in [26], Wright proposed a system for decision-making and operations on a fully autonomous ship using machine learning and artificial intelligence with multiple sensor modalities.

### 2.3. Summary

Recent studies have considered constraints such as network connectivity and energy consumption. All coverage formulas assume that the sensor has a given detection range in event perception methods or assume the distribution of sensor measurement values in the appropriate perception method; examples include [27] for wind monitoring and [28] for data center server overheating detection. To design deployment methods, considering the characteristics of application instances, a new application-aware deployment method shown in Table 1 is proposed. The thermal sensor deployment in this paper is similar. In previous studies, methods of identifying the most informative sensors have been proposed and implemented in several fields. Several predictive models and their distribution have been discussed. In this research, the data only contained the temperature for each site on each day. Thus, one site can be the dependent variable, and another site can be the independent variable. Correlations between the dependent and independent variables can be analyzed to discover correlative pairs. Due to cost or energy consumption considerations, the solution is proposed to reduce the number of deployed sensors and yield results within the error tolerance. The correlation-aware method is also designed in a mathematical model that combines theoretical and practical perspectives to determine sensor deployment strategies.

Summary of the Research Gap:

- Focuses on the temperature data which can be widely applied in various fields, such as humidity monitoring, air quality sensing, GPS surveillance, or landslide detection.
- Uses optimization-based methods to achieve reliable results.
- The methods used in this work are suitable for data from other fields.

## 3. System Architecture and Problem Formulation

### 3.1. System Structure

Characterization of the Deployment Region: IoT applications have recently expanded to almost every industry. To increase flexibility, we propose an optimal deployment strategy of minimizing cost while ensuring the accuracy of the collected data. Temperature data were used in the mathematical model, but the research can also be applied to other data such as humidity, air quality, or pressure [1,2,4]. Therefore, we attempt to generalize the mathematical model for sensor deployment. The mathematical model is suitable for application in different time zones or different climates.

Node Deployment: ACORN-SAT dataset includes consistent and uniform daily temperature records from 112 observation sites beginning in 1910. Figure 1b presents all the sites. The website (http://www.bom.gov.au/climate/data/acorn-sat/ (accessed on 20 October 2021).) displays ACORN-SAT v.2 (1911–2019), which is the original record of each highest and lowest daily temperature without any modifications due to extreme weather or strange weather records. Training data were collected from year of 2010–2018 and the testing data from 2019. The dataset contains relevant data including the longitude and the latitude of each site; the data were thus separated into different sections by longitude. These data-sets were used to verify the suitability of the mathematical model in different time zones. We propose three categories of experiments: (1) all 112 sites are used in one topology, (2) the sites are separated into two equal areas by longitude, and (3) the sites are separated into four equal areas by longitude.

**Figure 2:**
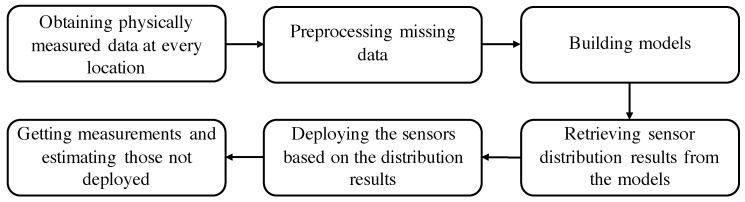
Flowchart of proposed solution approach.

Methodology Design: In Figure 2, the solution approach was proposed. Data were first collected from deployed sensors. However, some data loss may have occurred due to network processing, causing missing values in the obtained data. For example, in 2019, the temperature data for Tennant Creek, NT, has six days of missing data; and 18 days of missing data for Bridgetown, WA. Overall, there are 1% of missing data. These missing values were determined through interpolation. The intact data were then input into the four proposed solution models: Pearson correlation coefficients (PCC) and linear regression, XGBoost, referencing capability ranking (RCR), and *x* coefficient ranking (xCR). Finally, the Lagrangian Relaxation (LR) model was used to determine gaps between the objective cost and the theoretical minimum for each model.

### 3.2. Problem Formulation

In the proposed mathematical formulation, *D* is the set of evaluation data obtained before applying the model. The data in *D* are the ground truth of whether a sensor is installed at each location (i.e., all measured temperature values of all locations for all time zones). The main notations used in this study are presented in Table 2 and Table 3. The decision variables express the experimental outcome and indicate the sensor distribution and corresponding parameters.

The objective function of cost minimization is expressed by Equation (1). The total cost of deployment is the sum of the cost of the installed sensors. Variables *i* and *j* specify the locations of sensor nodes and $x_{i}$ is Boolean and indicates whether a sensor is installed at location *i*.

The mathematical model is based on the convection of heat energy and the conduction of radiation between points. Consequently, each temperature value is related to the other. By determining the relevance of the temperature of each location at each time, we can estimate temperatures at locations without sensors using the accurate measurements of the surrounding. We denote the estimated temperature at location *i* in dataset *k* to be *m*. Each solution model identifies *m* in different ways as discussed in Section 4. A universal method of calculating error is used in each method (Equation (3)). The overall error threshold of the system is expressed by Equation (4).

Solution models were primarily evaluated by minimizing $e_{ki}$ in Equation (3). The LR method was used to iteratively achieve the primal feasible outcome, i.e., the lowest deployment cost. The objective function of the primal problem is presented in Equation Integer Programming (IP) subject to constraints (2)∼(7).

(1) $$\begin{array}{ccc} \; & \min\;\sum_{i\in V}C_{i}x_{i} & \forall i\in V \end{array}$$

s.t.

(2) $$\begin{array}{ccc} \; & m_{ki}=\sum_{j\in V}p_{ji}H_{kj}x_{j} & \;\;\forall k\in D,\forall i,j\in V,j\neq i \end{array}$$

(3) $$\begin{array}{ccc} \; & e_{ki}={\left(m_{ki}-H_{ki}\right)}^{2} & \;\;\;\forall k\in D,\forall i\in V \end{array}$$

(4) $$\begin{array}{cc} \; & T=\sum_{k\in D}\sum_{i\in V}\frac{W_{i}e_{ki}}{\left|D||V\right|}\leq \Psi \end{array}$$

(5) $$\begin{array}{ccc} \; & \varepsilon \leq p_{ji}\leq \Pi_{ji} & \;\;\;\;\forall i,j\in V \end{array}$$

(6) $$\begin{array}{ccc} \; & x_{i}\in \left\{\varepsilon ,1\right\} & \;\;\;\;\forall i\in V \end{array}$$

(7) $$\begin{array}{ccc} \; & 0\leq e_{ki}\leq Z_{ki} & \;\;\;\forall k\in D,\forall i\in V. \end{array}$$

## 4. Solution Approach

### 4.1. Lagrangian Relaxation-Based Method

**Figure 3:**
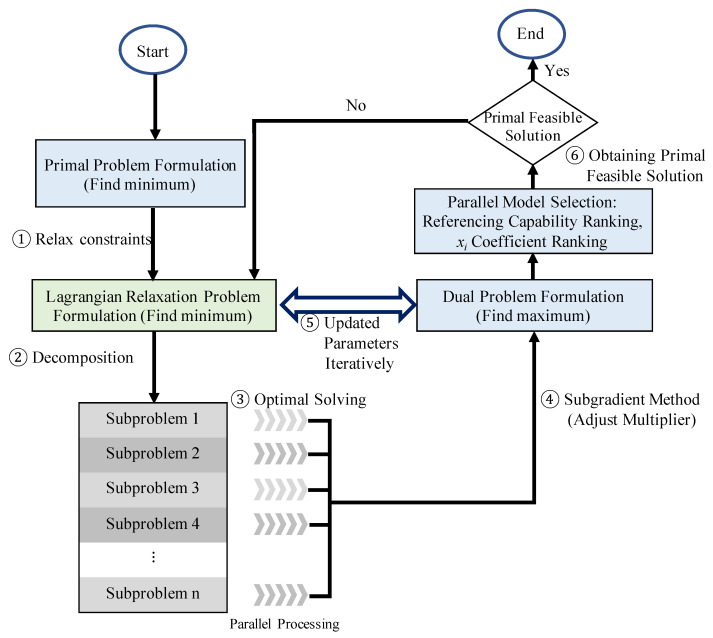
Procedures of the Lagrangian relaxation-based approach.

Lagrangian relaxation-based solution approach was widely studied in the 1970s. The LR problem can be established by removing the complex constraints and appending them after identifying the objective function with weights. The weights are Lagrangian multipliers and symbolize penalties when constraints are broken [29]. In this paper, the objective is to obtain the solution to the primal problem. The algorithm is followed by the procedures of the Lagrangian relaxation-based approach shown in Figure 3. Based on the mathematical formulation, the LR problem can be solved by disintegration to several independent subproblems. The LR problem is divided into five subproblems to find minima. Each subproblem is optimally solved using a divide-and-conquer approach. If a minimization problem is considered, the solution of the LR approach is the lower bounds [30]. The lower bounds are improved by adjusting the multipliers set between the LR and the dual problems. After obtaining a solution of the dual problem, its feasibility must be further checked or adjusted by the proposed and self-designed heuristics, such as RCR, xCR, or xGBoost (parallel model selections) for obtaining the primal feasible solution. A solution is feasible if it satisfies all constraints of the primal problem. The answer is marked if there is a feasible solution determined by a feasible check. Finally, the gap between the lower bounds and the feasible solutions is calculated for the entire process. The calculations are iteratively repeated until the termination conditions are satisfied.

#### 4.1.1. Step 1: Reformulation for Relaxation

Complicated constraints are relaxed to obtain a primal optimization problem and feasible solution regions are extended to simplify the primal problem. The primal problem is then transformed into an LR problem associated with Lagrangian multipliers. In accordance with the decomposition of decision variables, $p_{ji}x_{j}$, we introduce an auxiliary variable, $s_{ji}$ to reformulate the constraint equation (Equation (2)) by replacing $p_{ji}x_{j}$ with $s_{ji}$ to form the constraint Equations (8)–(10).

(8) $$\begin{array}{ccc} \; & \log s_{ji}=\log p_{ji}+\log x_{j} & \;\;\forall i,j\in V,j\neq i \end{array}$$

(9) $$\begin{array}{ccc} \; & m_{ki}=\sum_{j\in V}H_{kj}s_{ji} & \;\;\forall k\in D,\forall i,j\in V,j\neq i \end{array}$$

(10) $$\begin{array}{ccc} \; & \varepsilon \leq s_{ji} & \;\;\forall i,j\in V,j\neq i \end{array}$$

#### 4.1.2. Steps 2 and 3: Decomposition and Solution of Subproblems

We then relax the reformed mathematical model. The constraint Equations (3), (4), (8), and (9) were relaxed by introducing the Lagrange multipliers $\mu_{ki}^{1}$, $\mu^{2}$, $\mu_{ki}^{3}$, and $\mu_{ki}^{4}$. Consequently, the original problem is transformed into the LR problem as the objective function in Equation (11) subject to constraints (5), (6), (7), and (10).

(11) $$\begin{array}{cc} \; & \min\;Z_{LR}=\sum_{i\in V}C_{i}x_{i}\\ \; & +\sum_{k\in D}\sum_{i\in V}\mu_{ki}^{1}\left[e_{ki}-{\left(m_{ki}-H_{ki}\right)}^{2}\right]\\ \; & +\mu^{2}\left[\sum_{k\in D}\sum_{i\in V}\left(\frac{W_{i}e_{ki}}{\left|V\right|}\right)-\Psi \right]\\ \; & +\sum_{k\in D}\sum_{i,j\in V,j\neq i}\mu_{ki}^{3}\left[\log p_{ji}+\log x_{j}-\log s_{ji}\right]\\ \; & +\sum_{k\in D}\sum_{i\in V}\mu_{ki}^{4}\left[\sum_{j\in V,j\neq i}H_{kj}s_{ji}-m_{ki}\right]\\ \; & s.t.\;\;(5),\;(6),\;(7),\;\mathrm{and}\;(10). \end{array}$$

The LR problem is then decomposed into five subproblems. The objective of this step is to reach a minimum $Z_{LR}$. Thus, each subproblem is solved individually. Each minimum is obtained by algorithms for the five subproblems determining $s_{ji}$, $x_{i}$, $p_{ji}$, $e_{ki}$, and $m_{ki}$ are presented as follows.

- **Subproblem 1** (related to $s_{ji}$)
  (12) $$\begin{array}{cc} \; & \min\;\sum_{k\in D}\sum_{i,j\in V,i\neq j}\left(-\mu_{ki}^{3}\log s_{ji}+\mu_{ki}^{4}H_{kj}s_{ji}\right)\\ \; & s.t.\;\;\varepsilon \leq s_{ji}\leq \Pi_{ji}\;\forall i,j\in V,i\neq j. \end{array}$$
  Subproblem 1 is a minimization problem. It consists of continuous variable and logarithm, extremum will be found when differential equals zero or the boundaries of the decision variable, $S_{ji}$. Algorithm 1 shows the pseudo-code of Subproblem 1. First, we calculate the partial differential of the objective function by $S_{ji}$. Let the product is equal to zero to determine the value of $\frac{\mu_{ki}^{3}}{\mu_{ki}^{4}H_{kj}}$. Secondly, the validity of the extremum must be checked. Setting the $S_{ji}$ equal to ε, $\frac{\mu_{ki}^{3}}{\mu_{ki}^{4}H_{kj}}$, or $\Pi_{ji}$ such that the objective value of (Sub 1) is minimum, correspondingly.
**Algorithm 1** Subproblem 1**Input:** Given parameters H and Lagrangian multipliers $\mu^{3}$, $\mu^{4}$.**Output:** Decision variable s.**Initialize:**$s_{ji}\leftarrow 0$, ∀i,j∈V,i≠j**for**k=0 to (|D|−1) **do**      **for** j=0 to (|V|−1) **do**            **if** i=j **then**                continue            **end if**            **if** $\varepsilon \leq \frac{\mu_{ki}^{3}}{\mu_{ki}^{4}H_{kj}}\leq \Pi_{ji}$ **then**                $s_{ji}\leftarrow \varepsilon$, $\frac{\mu_{ki}^{3}}{\mu_{ki}^{4}H_{kj}}$, or $\Pi_{ji}$ such that $-\mu_{ki}^{3}\log s_{ji}+\mu_{ki}^{4}H_{kj}s_{ji}$ has minimum.            **else**                $s_{ji}\leftarrow \varepsilon$ or $\Pi_{ji}$ such that $-\mu_{ki}^{3}\log s_{ji}+\mu_{ki}^{4}H_{kj}s_{ji}$ has minimum.            **end if**      **end for****end for**
- **Subproblem 2** (related to $x_{i}$)
  (13) $$\begin{array}{cc} \; & \min\;\sum_{i,j\in V,i\neq j}\left(C_{i}x_{i}+\sum_{k\in D}\mu_{ki}^{3}\log x_{j}\right)\\ \; & s.t.\;\;x_{i}\in \left\{\varepsilon ,1\right\}\;\forall i\in V. \end{array}$$
  Subproblem 2 is further decomposed into |V| independent minimization problems. For a location *i*, the decision variable $x_{i}$ is examined for two values which are ε or 1, such that $C_{i}x_{i}+\mu_{\mathrm{sum}}\log x_{i}$ has minimum, respectively. The pseudo-code is shown in Algorithm 2.
**Algorithm 2** Subproblem 2**Input:** Given parameters C and Lagrangian multiplier $\mu^{3}$.**Output:** Decision variable x.**Initialize:**$x_{i}\leftarrow 0$, ∀i∈V**for**i=0 to (|V|−1) **do**     $\mu_{\mathrm{sum}}\leftarrow 0$     **for** k=0 to (|D|−1) **do**           **for** j=0 to (|V|−1) **do**               **if** j=i **then**                 continue               **else**                 $\mu_{\mathrm{sum}}\leftarrow \mu_{\mathrm{sum}}$ + $\mu_{kj}^{3}$               **end if**           **end for**     **end for**     $x_{i}\leftarrow \varepsilon$ or 1 such that $C_{i}x_{i}+\mu_{\mathrm{sum}}\log x_{i}$ has minimum.**end for**
- **Subproblem 3** (related to $p_{ji}$)
  (14) $$\begin{array}{cc} \; & \min\;\sum_{k\in D}\sum_{i,j\in V,i\neq j}\mu_{ki}^{3}\log p_{ji}\\ \; & s.t.\;\;\varepsilon \leq p_{ji}\leq \Pi_{ji}\;\forall i,j\in V,i\neq j. \end{array}$$
  Subproblem 3 is also a minimization problem consisting continuous variable and logarithm. Since (Sub 3) is a logarithmic equation, the minimum of such equation lies at either one of the boundaries of $p_{ji}$. Therefore, Algorithm 3 simply checks either ε or $\Pi_{ji}$ has the minimum value. Algorithm 3 shows the pseudo-code of Subproblem 3.
**Algorithm 3** Subproblem 3**Input:** Given Lagrangian multiplier $\mu^{3}$.**Output:** Decision variable p.**Initialize:**$p_{ji}\leftarrow 0$, ∀i,j∈V,i≠j**for**k=0 to (|D|−1) **do**      **for** i=0 to (|V|−1) **do**         **for** j=0 to (|V|−1) **do**            **if** j=i **then**               continue            **else**               $p_{ji}\leftarrow \varepsilon$ or $\Pi_{ji}$ such that $\mu_{ki}^{3}\log p_{ji}$ has minimum.            **end if**        **end for**      **end for****end for**
- **Subproblem 4** (related to $e_{ki}$)
  (15) $$\begin{array}{cc} \; & \min\;\sum_{k\in D}\sum_{i\in V}\left(\mu_{ki}^{1}+\mu^{2}\frac{W_{i}}{\left|V\right|}\right)e_{ki}\\ \; & s.t.\;\;0\leq e_{ki}\leq \max\left(H_{ki}\right)-\min\left(H_{ki}\right)\;\forall k\in D,\forall i\in V. \end{array}$$
  Subproblem 4 aims at deriving the right $e_{ki}$ such that $\left(\mu_{ki}^{1}+\mu^{2}\frac{W_{i}}{\left|V\right|}\right)e_{ki}$ has minimum. Since $\mu_{ki}^{1}+\mu^{2}\frac{W_{i}}{\left|V\right|}$ is given, $e_{ki}$ can be determined by checking whether $\mu_{ki}^{1}+\mu^{2}\frac{W_{i}}{\left|V\right|}$ is negative. Algorithm 4 shows the pseudo-code of Subproblem 4.
**Algorithm 4** Subproblem 4**Input:** Given parameters W and Lagrangian multipliers $\mu^{1}$, $\mu^{2}$.**Output:** Decision variable e.**Initialize:**$e_{ki}\leftarrow 0$, ∀k∈D,∀i∈V**for**k=0 to (|D|−1) **do**      **for** i=0 to (|V|−1) **do**         **if** $\mu_{ki}^{1}+\frac{\mu^{2}W_{i}}{\left|V\right|}\geq 0$ **then**            $e_{ki}\leftarrow 0$         **else**            $e_{ki}\leftarrow {\left(\max\left(H_{ki}\right)-\min\left(H_{ki}\right)\right)}^{2}$         **end if**      **end for****end for**
- **Subproblem 5** (related to $m_{ki}$)
  (16) $$\begin{array}{cc} \; & \min\;\sum_{k\in D}\sum_{i\in V}\left[-\mu_{ki}^{1}m_{ki}^{2}+\left(2\mu_{ki}^{1}H_{ki}-\mu_{ki}^{4}\right)m_{ki}\right]\\ \; & s.t.\;\;\min\left(H_{ki}\right)\leq m_{ki}\leq \max\left(H_{ki}\right)\;\forall k\in D,\forall i\in V. \end{array}$$
  Subproblem 5 is a quadratic equation of $m_{ki}$, so the minimum could be found by its differential. The boundary of $m_{ki}$ lies between $\min(H_{ki})$ and $\max(H_{ki})$. Algorithm 5 first checks whether the differential $\frac{2H_{ki}\mu_{ki}^{1}-\mu_{ki}^{4}}{2\mu_{ki}^{1}}$ lies within the boundary. If so, the $m_{ki}$ should be either $\min(H_{ki})$, $\max(H_{ki})$, or $\frac{2H_{ki}\mu_{ki}^{1}-\mu_{ki}^{4}}{2\mu_{ki}^{1}}$. Otherwise, the minimum happens at the one of boundaries, so $m_{ki}$ should be either $\min(H_{ki})$ or $\max(H_{ki})$. Algorithm 5 shows the pseudo-code of Subproblem 5.
**Algorithm 5** Subproblem 5**Input:** Given parameters H and Lagrangian multipliers $\mu^{1}$, $\mu^{4}$.**Output:** Decision variable m.**Initialize:**$m_{ki}\leftarrow 0$, ∀k∈D,∀i∈V**for**k=0 to (|D|−1) **do**      **for** i=0 to (|V|−1) **do**         **if** min$\left(H_{ki}\right)\leq \frac{2H_{ki}\mu_{ki}^{1}-\mu_{ki}^{4}}{2\mu_{ki}^{1}}\leq$ max$\left(H_{ki}\right)$ **then**            $m_{ki}\leftarrow \min\left(H_{ki}\right)$, $\frac{2H_{ki}\mu_{ki}^{1}-\mu_{ki}^{4}}{2\mu_{ki}^{1}}$, or $\max(H_{ki})$ such that $\mu_{ki}^{1}\left(-m_{ki}^{2}+2m_{ki}H_{ki}\right)-\mu_{ki}^{4}m_{ki}$ has minimum.         **else**            $m_{ki}\leftarrow \min\left(H_{ki}\right)$ or $\max(H_{ki})$ such that $\mu_{ki}^{1}\left(-m_{ki}^{2}+2m_{ki}H_{ki}\right)-\mu_{ki}^{4}m_{ki}$ has minimum.         **end if**      **end for****end for**

#### 4.1.3. Steps 4 and 5: Dual Problem and Subgradient Method

The LR problem can be solved optimally if all subproblems are solved optimally using the divide-and-conquer approach. The optimal value of the LR problem, denoted as $Z_{LR}$, is an LB of $Z_{IP}$. Hence, to derive the LB, we must adjust the Lagrangian multipliers to identify those with the greatest values by solving the dual problem shown in (17).

(17) $$\begin{array}{cc} \; & \max Z_{D}=Z_{LR}\left(\mu_{ki}^{1},\mu^{2},\mu_{ki}^{3},\mu_{ki}^{4}\right)\\ \; & s.t.\;\;\mu_{ki}^{1},\mu_{ki}^{3},\mu_{ki}^{4}\in R,\mu^{2}\geq 0\;\forall k\in D,\forall i\in V. \end{array}$$

The subgradient method proposed by Held and Karp [31,32] is a commonly used approach for solving the dual problem due to the simplicity of its programming. First, we let vector *m* be a subgradient of the dual problem. Over *n* iterations of the subgradient method, the multiplier vector is updated by Equation (18).

(18) $$\mu_{n+1}=\mu_{n}+t_{n}m_{n}$$

(19) $$t_{n}=\lambda_{n}\frac{\left[Z_{IP}-Z_{D}\left(\mu_{n}\right)\right]}{\left|\right|m_{n}{\left|\right|}^{2}}$$

The step size $t_{n}$ is defined in Equation (19). According to the work of Held et al. [33], $\lambda_{n}$ is a scalar. Usually, it is set to two and halved if $Z_{D}\left(\mu_{n}\right)$ cannot increase within a certain number of iterations. The procedures of LR method and the subgradient method are presented in Figure 3. It is the way to find the tightest lower bound of the dual problem iteratively.

#### 4.1.4. Step 6: Obtaining the Primal Feasible Solutions

A set of decision variables was extracted after the five subproblems were solved. However, due to the relaxation of multiple complex constraints, the solution may not be feasible (as mentioned in Section 4.1). Therefore, we designed heuristic methods to tune decision variables to achieve feasibility. The two proposed methods were RCR and xCR.

- **Referencing Capability Ranking (RCR)**
  The primary goal of this study was to accurately estimate temperature measurement at locations without sensors. Equation (2) describes the estimation model; where $m_{ki}$ can be calculated by a linear combination of the data series $H_{kj}$ and coefficients $p_{ji}$. Thus, the problem is reduced to deriving an optimal series of $p_{ji}$ such that $e_{ki}$ in Equation (3) is minimized. To derive $p_{ji}$, we apply the steepest gradient descent method. The steepest gradient descent method, also known as the gradient method, was first described by Cauchy in 1847. Other analytic methods have been inspired by the method or derived from its deformation; the gradient method is thus fundamental to optimization methods. The method requires minimal work and few storage variables, and has low initial point requirements. However, it converges slowly, is inefficient, and sometimes is unable to yield an optimal solution. The goal of nonlinear programming is the numerical optimization of nonlinear functions. The theory and methods of nonlinear programming are used in military, economic, management, production process automation, engineering design and product optimization design applications. Nonlinear programming methods were used to calculate the optimal set of the coefficient $p_{ji}$ in Equation (2). The objective function for gradient descent is Equation (20). In each LR iteration, the $x_{i}$ of each subproblems is used to optimize the corresponding $p_{ji}$. After several rounds of gradient descent, if the overall error of estimated measurements using optimized $p_{ji}$ satisfies the average error threshold Ψ the set $x_{i}$ is considered feasible and the total cost is recorded. However, if Ψ is not satisfied, locations for deployed sensors are added to reduce the overall error. The addition is based on the ranking of $f_{i}$ of each location *i*; $f_{i}$ is the “referencing ability” of location *i* for other locations as presented in Equation (21).
  (20) $$\min\;\sum_{k\in D}\sum_{i\in V}{\left(m_{ki}-H_{ki}\right)}^{2}$$
  (21) $$f_{i}=\frac{\sum_{j\in V}p_{ji}}{C_{i}},\;\forall i\in V.$$
- ***x* Coefficient Ranking (xCR)**
  xCR is similar to RCR. In this strategy, Equation (2) was also applied to estimate $m_{ki}$ given $x_{i}$ by the LR procedure in each iteration. Moreover, steepest gradient descent was used to determine $p_{ji}$ to minimize objective error (Equation (20)). If, after several rounds of gradient descent, if the overall estimated measurement error does not satisfy the average error threshold Ψ, locations are added to mitigate the error based on the ranking of the coefficients of $x_{i}$ in the LR objective formulation until a feasible set of $x_{i}$ is produced.

**Figure 4:**
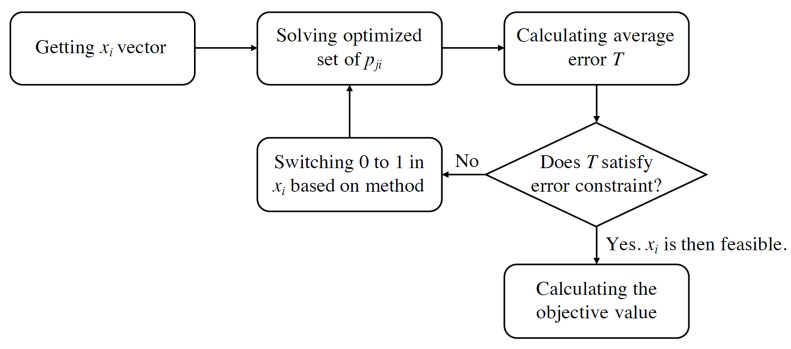
Procedures of the Lagrangian relaxation-based method for obtaining feasible solutions.

In summary, to achieve a feasible solution set $x_{i}$ and its objective value (Equation (1)), $x_{i}$ from Algorithm 1 is first used to calculate the optimized set of $p_{ji}$. Then, the average error of estimation *T* is compared with the threshold Ψ. If the threshold is satisfied, the set $x_{i}$ is considered feasible. Otherwise, some *i* in $x_{i}$ are changed from zero to one (i.e., sensors are installed) based on either the RCR or xCR method to mitigate error. This process is illustrated in Figure 4.

### 4.2. Pearson Correlation Coefficients and Linear Regression Methods

The PCC model begins with determining its coefficients:

(22) $$c_{ji}=\frac{cov(H_{ki},H_{kj})}{\sigma_{H_{ki}}\sigma_{H_{kj}}}$$

(23) $$-1\leq c_{ji}\leq 1\;\forall i,j\in V.$$

The value of $c_{ji}$ is the Pearson correlation coefficient. The term cov in Equation (22) refers to the covariance between $H_{ki}$ and $H_{kj}$ where the data at location i,j∈V are all included in the dataset *D*. The term σ is the standard deviation of variables $H_{ki}$ and $H_{kj}$; the data at location i,j∈V are all included in the dataset *D*. Each pair of data at location *i* and *j* in dataset *D* have the same value regardless the order of *i* and *j*. If *i* and *j* are the same in the equation, the value of $c_{ji}$ is 1, indicating identical data pairs. If the value of $c_{ji}$ is between ±0.5 and ±1.0, the correlation is considered strong. The values of $c_{ji}$ between ±0.3 and ±0.49 indicate moderate correlation. Values of $c_{ji}$ lower than ±0.29 indicate weak correlation between *i* and *j*. Negative $c_{ji}$ implies that the data are negatively correlated. Thus, pairs with greater $|c_{ji}|$ have stronger correlations.

Because our goal is to minimize the cost of sensor deployments, the temperature value of locations without installed sensors must be estimated. In PCC, for any measuring location without a sensor, the measured values of several nearby sensors are required to estimate a value for the missing sensor through linear regression. The correlation coefficient obtained through convex combination is a decision variable. Zero indicates no association. Each location without a sensor is associated with all measured values at locations with installed sensors.

The estimated measurement value $m_{kj}$ at location *j* can be obtained from Equation (24) for location j∈V and dataset k∈D. $H_{ki}$ is the measurement value physically collected from sensors. After calculation of the correlation coefficients, the relationships between each pair *i* and *j* are all known. Thus, rankings of the correlation coefficients can indicate sensor deployment locations. In Equation (24), coefficients $a_{ji}$ and $b_{ji}$ are obtained from the training data-sets $H_{ki}$ and $H_{kj}$. $H_{ki}$ indicates the independent variable and $H_{kj}$ is the dependent variable. After obtaining $a_{ji}$ and $b_{ji}$, the testing data $H_{ki}$ can be used to obtain a predicted value $m_{kj}$. In short, the measurement at location *j* is inferred using an actual measurement at location *i*. If the correlation coefficient of a selected pair is positive, then the corresponding $a_{ji}$ is also positive. However, if data for the independent variable and dependent variable is switched, both $a_{ji}$ and $b_{ji}$ will change. The best solution will have a lower residual for the result of the equation; that is, the coefficient of determination ($R^{2}$) will be higher. Because Equation (24) is a unary linear regression, $R^{2}$ of the selected pair equals the square of the correlation coefficient. Therefore, the correlation coefficient can be used to determine the value of $R^{2}$ for a given pair.

(24) $$m_{kj}=a_{ji}H_{ki}+b_{ji}$$

∀k∈D,∀i,j∈V,i≠j

Because the Pearson correlation coefficient is a measurement of the strength of the association between two variables, high coefficients for two given nodes have two meanings:

- The node is strongly associated with other nodes; the temperature can thus be accurately estimated by other nodes.
- The node is strongly associated with other nodes; it can be used to estimate temperatures at other nodes.

To decide where to deploy a sensor, the summation of the correlation coefficients for each site can be ranked to determine each node’s connectivity. Strongly connected nodes are preferentially installed.

**Figure 5:**
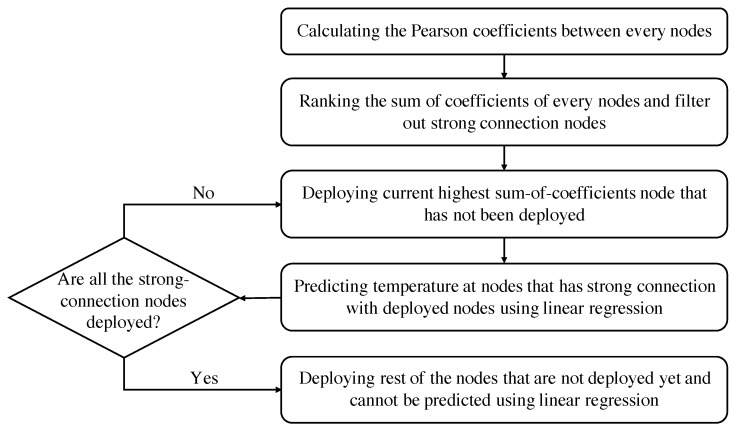
Procedures of Pearson correlation coefficient method for sensor deployment.

The node with the highest sum of coefficients is first deployed. Then, the temperature at all other nodes connected to the deployed node can be predicted using linear regression; deployment of these nodes can be avoided. Next, the node with the second-highest sum of coefficients is deployed, and nodes strongly connected with this deployed node are not deployed. This process continues until all strongly connected nodes are deployed or have been removed. Finally, leftover nodes are deployed because they are not predicted by any deployed node. The algorithm is presented in Figure 5.

### 4.3. Extreme Gradient Boosting Method

Extreme gradient boosting (XGBoost) is a decision-tree-based boosting system that is well known and widely used in machine learning [34,35]. The system can meet the classifying and regression requirements of our method. Assuming *K* trees in the classification, *F* denotes the space of functions containing all regression trees, $f_{k}\left(x_{i}\right)$ is the weight of the $i^{th}$ sample in the $k^{th}$ tree. The model is formulated by Equation (25):

(25) $$m_{ki}=\sum_{k=1}^{K}f_{k}\left(x_{i}\right)\;,\forall f_{k}\in F$$

In the XGBoost model, we split the data 8:2 for the training set and testing set. The main objective of using XGBoost is to train a model that can estimate the temperature at sensor-uninstalled locations. In each iteration of LR, the subproblems produce a random set of $x_{i}$ indicating whether a sensor is installed at location *i*. XGBoost then trains itself using this set of $x_{i}$. After completing training, the model verifies whether the overall error of estimated measurements is below the average error threshold Ψ. If so, the set $x_{i}$ is considered feasible and its total cost is recorded. Otherwise, the set $x_{i}$ is discarded and the model proceeds to the next iteration.

## 5. Computational Experiments

**Figure 6:**
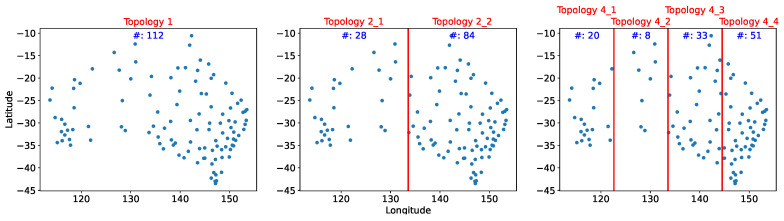
Sensor distribution in the three distinct topologies.

We compared different methods and topologies within the system tolerance on the average estimation error. Ψ is set to temperature 1.5 degrees. The sensors were divided into one, two, and four equal area clusters by longitude. Because the sensors were not uniformly distributed, the eastern section contained more sensors; Topology 2_2 had twice as many sensors as Topology 2_1. Figure 6 depicts the distribution and number of sensors for each topology.

The experiments were conducted using a computer with an AMD Ryzen 5 5600X 6-Core Processor @3.7 GHz, 32 GB RAM, and under Windows 10 Professional 19041.1165 and Python 3.7.6.

The experimental parameters are listed in Table 4. Table 5 displays the experimental outcomes for each optimization method and topology. The table reveals that the XGBoost, RCR, and xCR methods were more effective in finding minimum deployment costs than the PCC method was. PCC typically deployed more sensors than other methods. Furthermore, the performance of XGBoost, RCR, and xCR did not differ substantially.

### 5.1. XGBoost

**Figure 7:**
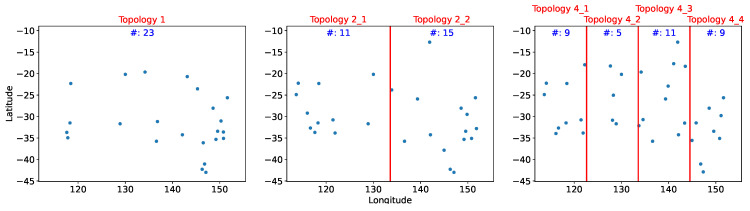
Sensor deployment distribution from XGBoost.

Figure 7 displays the results of deployment using XGBoost. The distribution of the sensor nodes was less dense and the sensors were more evenly distributed across the region compared with the distribution in Figure 6 where sensors are densely situated in the southeast region. In particular, for Topology 4, sensors were more evenly distributed than in Topology 1 or Topology 2. The easternmost section (Topology 4_4) included exactly the same number of sensors as in the westernmost section (Topology 4_1).

**Figure 8:**
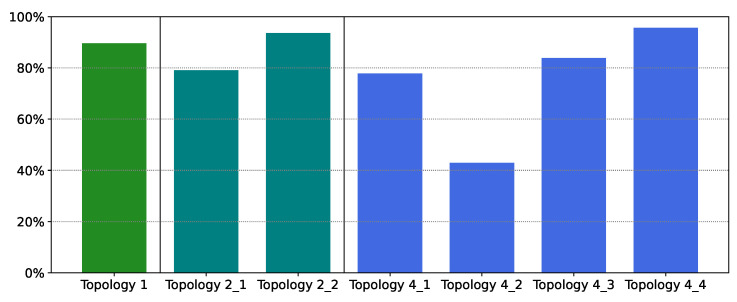
Cost reduction for each topology using XGBoost.

Figure 8 displays the cost reduction for each method compared with the original cost. The performance of XGBoost is outstanding; costs have been reduced by 80% on average, compared to the case when all 112 sensors are distributed. Even in the worst case (Topology 4_2) the cost has been reduced by over 40%. Moreover, Figure 8 also reveals that the cost reduction is primarily from sensor reduction in the eastern region; that is, savings were greater for Topology 2_2 and Topology 4_4 compared with their western counterparts.

### 5.2. RCR

**Figure 9:**
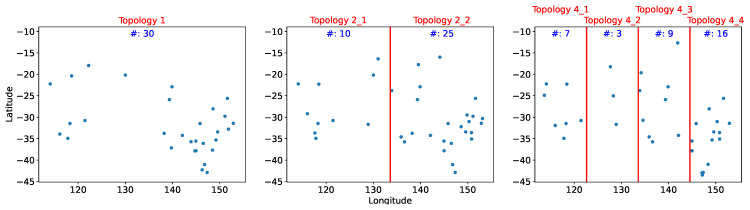
Sensor deployment distribution from referencing capability ranking.

Sensor distributions obtained using LR with the RCR heuristic method of identifying feasible solutions are displayed in Figure 9. Table 5 reveals that the performance of RCR was similar to that of XGBoost for both overall cost and number of sensors used. Cost reduction was also approximately 80% on average. However, the sensor distribution differed between these two methods. Figure 9 reveals that sensors are still unevenly distributed over the territory; Topology 2_2 has twice as many sensors as Topology 2_1. Topology 4_4 also had at least twice as many sensors as Topology 4_1, Topology 4_2, and Topology 4_3.

### 5.3. xCR

**Figure 10:**
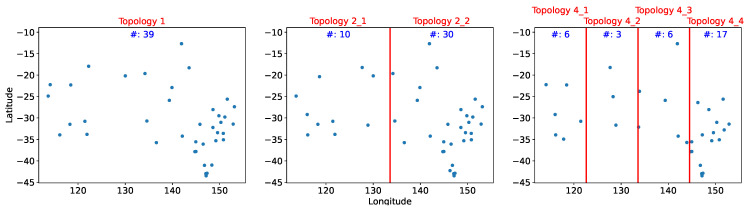
Sensor deployment distribution from *x* Coefficient Ranking.

Sensor distributions obtained using LR with the xCR heuristic method of identifying feasible solutions are displayed in Figure 10. The performance of xCR was also similar to that of XGBoost and RCR. However, as revealed by Figure 10, the sensor distribution is substantially more concentrated in the southeast region compared with those of XGBoost and RCR. The two overlapping sensors at the most southeastern are of Topology 2_2 and Topology 4_4 are both in Tasmania at location 42.89${}^{\circ }$ S, 147.33${}^{\circ }$ E and 42.99${}^{\circ }$ S, 147.07${}^{\circ }$ E; the distance between these sensors is only 23.95 km.

### 5.4. Pearson Correlation and Linear Regression

**Figure 11:**
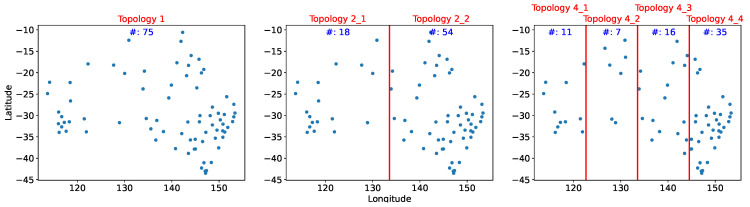
Sensor deployment distribution from Pearson correlation coefficients.

**Figure 12:**
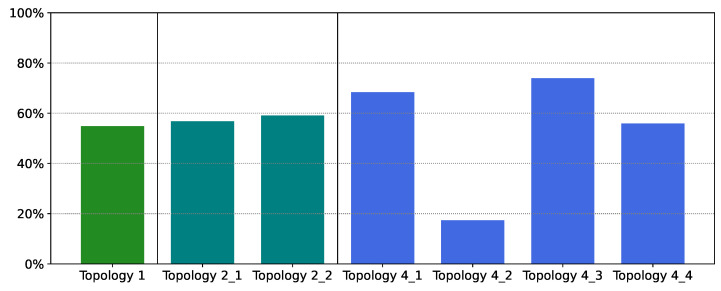
Cost reduction for each topology using Pearson correlation coefficients.

Table 5 presents the total cost and number of sensors deployed using PCC; the number of sensors is significantly greater than that of other methods. Figure 11 reveals that the sensors are again densely situated in the southeast region; few sensors have been eliminated compared with the distribution in Figure 6. Figure 12 demonstrates that cost reduction from PCC was only 55% on average across the topologies; this result was substantially worse than the 80% cost reduction of XGBoost. For example, in Topology 4_2 PCC removes only one sensor; cost reduction in that region was only 17%.

### 5.5. Extended Application

#### 5.5.1. Lifetime Enhancement

The preceding experiments were conducted under various topologies. However, some types of sensors require regular reinstallation; these sensors may have fixed lifetimes or nonrechargeable batteries. In this situation, deploying all sensors first followed by strategically activating sensors while predicting the measurements of inactive sensors within an error threshold may be desirable. After the active sensors exceed their lifetimes, inactive sensors can be strategically activated to predict the data of the previously active sensors. Maximizing the number of cycles could result in long sensor lifetimes with a fixed deployment cost.

The optimization model is applicable in this scenario. Because we aim to maximize the number of cycles, we must minimize the number of sensors in each cycle. Therefore, we can start from Topology 1, containing all 112 sensor nodes and find an optimal sensor distribution. Then, we can eliminate sensors which are out of battery and use the remaining sensors to find a new optimal distribution. This process can continue until all sensors have been used or the average error exceeds an error threshold.

**Figure 13:**
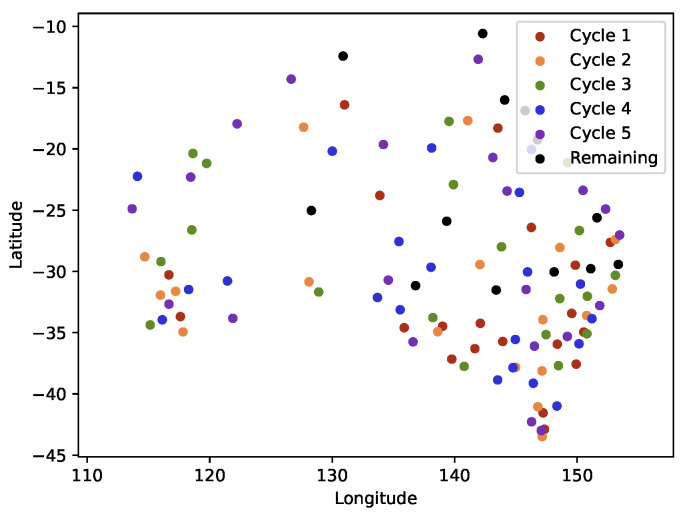
Active sensor distribution in each cycle.

In this lifetime enhancement experiment, XGBoost was used. The costs of all sensors were assumed to be identical to identify a minimum number of sensors in each cycle. Table 6 reveals the result; A maximum of five cycles can be achieved with approximately 20~sensors activated in each cycle without exceeding the error constraint. Thus, a one-time expenditure of deploying 99 sensors (the remaining 13 sensors were unnecessary) can be used for five times the lifetime of an individual sensor. The sensor distributions with colors for each cycle are depicted in Figure 13.

#### 5.5.2. Other Applications

The proposed model can be applied to not only temperature sensors but also humidity monitoring, air quality sensing, GPS surveillance, landslide detection [22,23,36,37,38,39], and other sensors. The ultimate goal of the model is to reduce deployment costs; the contribution of this study would be substantial for deployments with high sensing expenditures for other extreme or critical applications. Additionally, the solution could be used by deployment practitioners to enhance the lifetimes of sensors within error tolerance.

## 6. Conclusions

In this work, the goal was to minimize deployment costs for numerous sensors strategically. We chose the ACORN-SAT dataset to test the model. The dataset includes 112 sensor locations across Australia in ten years. The mathematical formulation is modeled and solved by the proposed procedures systematically. XGBoost, PCC, and the LR method using the heuristic RCR and xCR strategies were proposed and called error-bound satisfaction to determine the primal feasible solutions. Finally, we demonstrated that XGBoost and LR using RCR could reduce costs by 80%; thus, the goal was achieved. Furthermore, we introduced a method of using the model to maximize the lifetime of a sensor network which is sufficient to meet the requirements for controlling the sensors during operations. In conclusion, this work combines both theoretical and practical considerations to minimize the deployment cost of temperature sensors. The proposed solution can be readily applied to sensor distribution problems in various fields.

## Figures and Tables

**Table 1:** Proposed Model Comparisons With Literature.

Model	Related Work	Sensor Allocation	Cost/Energy Consumption	Data Collection	Correlation Aware	Deployment Strategy
Dynamic Coverage Measures	[9]		✓	✓	✓	
Sparsity-Enforcing Sensor Management Methods	[10]	✓				✓
Gaussian and Non-Gaussian Process	[12,27]	✓				✓
FrameSense	[14]	✓	✓	✓	✓	
Lightweight and Intelligent Intrusion Detection Method	[18]				✓	
Proposed model		✓	✓	✓	✓	✓

**Table 2:** Notations of Given Parameters.

Notation	Description
*D*	Index set of evaluation data, where D={1,2,…,k,…,|D|}
*V*	Index set of locations, where V={1,2,…,i,…,j,…,|V|}
$C_{i}$	Installation cost of sensor *i*, where i∈V
$H_{ki}$	Measurement is taken at location *i* in dataset *k*, where i∈V, k∈D
Ψ	Tolerance on the average estimation error
$W_{i}$	Weight is associated with location *i*, where i∈V ($0\leq W_{i}\leq 1$ and $\sum_{i\in V}W_{i}=1$)

**Table 3:** Notations of Decision Variables.

Notation	Description
$x_{i}$	Binary variable, 1 if sensor *i* is installed, and 0 otherwise
$p_{ji}$	Weighting factor is set from sensor *j* to estimate measurement at location *i*, where i,j∈V
$\Pi_{ji}$	Maximum weighting factor for sensor *j* to estimate measurement at location *i*, where i,j∈V
$m_{ki}$	Measurement is estimated for location *i* using dataset *k*, where i∈V, k∈D
$e_{ki}$	Estimation error is calculated at location *i* using dataset *k*, where i∈V, k∈D
$Z_{ki}$	Maximum estimation error calculated at location *i* using dataset *k*, where i∈V, k∈D
*T*	Average estimation error ($T=\sum_{k\in D}\sum_{i\in V}\frac{W_{i}e_{ki}}{\left|V||D\right|}$)

**Table 4:** Parameters for Computational Experiments.

Given Parameter	Value
Number of evaluation data (|D|)	3650
Number of locations (|V|)	112
$C_{i}$	5∼1000
$H_{ki}$	12.1 °C∼47.9 °C
Ψ	1.5 °C
$W_{i}$	0.000182∼0.018169, $\sum W_{i}=$ 1

**Table 5:** Experimental Outcomes for Each Method and Topol. (Topology).

Cost	Topol. 1	Topol. 2_1	Topol. 2_2	Topol. 4_1	Topol. 4_2	Topol. 4_3	Topol. 4_4
(# of Sensors)	(112)	(28)	(84)	(20)	(8)	(33)	(51)
XGBoost	6108	3186	2779	2210	3002	3096	1051
	(23)	(11)	(15)	(9)	(5)	(11)	(9)
RCR	8264	3732	6272	2157	2035	2288	2820
	(30)	(10)	(25)	(7)	(3)	(9)	(16)
*x*CR	7434	3858	5383	2073	2035	1904	3602
	(39)	(10)	(30)	(6)	(3)	(6)	(17)
PCC	26,382	6578	17,673	3149	4349	4994	10,620
	(75)	(18)	(54)	(11)	(7)	(16)	(35)

**Table 6:** Results of Sensor Network Lifetime Enhancement.

Cycle	1	2	3	4	5	Remaining
of Active Sensors	20	18	19	21	21	13

## Data Availability

Not applicable.
